# Supplementary material for: Postoperative radiotherapy timing, molecular subgroups and treatment outcomes of Thai pediatric patients with medulloblastoma
Source: PLoS One. 2023 Jan 17;18(1):e0271778. doi: 10.1371/journal.pone.0271778 (PMC9844848; doi:10.1371/journal.pone.0271778)
Supplement: S1 File — (PDF) [file pone.0271778.s001.pdf]

Supplementary data

Treatment protocol for medulloblastoma according to the Thai Pediatric Oncology Group (ThaiPOG) protocol.

All patients underwent maximal safe resection of the primary tumor followed by radiotherapy and chemotherapy. For infants age less than 36 months, neoadjuvant chemotherapy was given to delay RT for 1 year or when the patient reached 3 years of age, whichever comes first.

<u>Chemotherapy</u>

Chemotherapy during irradiation

- Vincristine 1.5 mg/m2 weekly x 6 weeks (max 2 mg/dose)

Chemotherapy after irradiation (start within 28 days after irradiation completion)

Cycle	Drug	Dose	Day
1,3,5,7,9	Cyclophosphamide	800 mg/m2/day IV drip in 1 hour daily	1-3
	Vincristine	1.5 mg/m2/day IV push (max 2 mg/dose)	1,8,15
2,4,6,8,10	Carboplatin	200 mg/m2/day IV drip in 1 hour daily	1-3
	Etoposide	150 mg/m2/day IV drip in 30 min<br>‟ 2 hr daily**	1-3

*New cycle of chemotherapy should be started within 21-28 days
*Chemotherapy should be started when ANC >1,000/ L and platelet >100,000/ L
*G-CSF will be given at 24-36 hr after completion of each course of chemotherapy
**A longer duration of administration may be used if the volume of fluid to be infused is a concern

Chemotherapy protocol for infants age less than 36 months
The protocol consists of cyclophosphamide, vincristine, methotrexate, carboplatin, and etoposide for a total duration of 52 weeks. The protocol will be finished at 1 year or the patient reaches 3 years of age, whichever comes first.

Cycle	Drug	Dose	Day
Week 1,10,19,28,37,46	Cyclophosphamide	800 mg/m^2/day IV drip in 1 hour daily	1-3
	Vincristine	1.5 mg/m^2/day IV push	1
Week 3,12,21,30,39,48	HDMTX	5 g/m^2/day IV drip in 4 hours	1
	Vincristine	1.5 mg/m^2/day IV push	1
Week 5,14,23,32,41,50	HDMTX	5 g/m^2/day IV drip in 4 hours	1
	Vincristine	1.5 mg/m^2/day IV push	1
Week 7,16,25,34,43,52	Carboplatin	200 mg/m^2/day IV drip in 1 hour daily	1-3
	Etoposide	150 mg/m^2/day IV drip in 30 min – 2 hr daily	1-3

- Start chemotherapy every 2 weeks, when ANC ≥ 750/µL and platelet ≥ 80,000/µL
- **Reduced dose of carboplatin and Vincristine according to patient's age**
  - age ≤ 6 months: use 66% of the full dose per BSA
  - age >6 months – 1 yr: use 80% of the full dose per BSA
  - age >1 year: use full dose of chemotherapy per BSA

Radiotherapy protocol

For average risk medulloblastoma
CSI 23.4-24 Gy (1.5-1.8 Gy/fraction) followed by whole posterior fossa radiation to at least 36 Gy and boost to tumor bed up to 54 - 55.8 Gy (1.8-2 Gy/fraction).

For high risk medulloblastoma
CSI 36 Gy (1.5-1.8 Gy/fraction) followed by either whole posterior fossa boost or a tumor bed boost up to 54-55.8 Gy (1.8-2 Gy/fraction).
