# Supplementary material for: Postoperative radiotherapy timing, molecular subgroups and treatment outcomes of Thai pediatric patients with medulloblastoma
Source: PLoS One. 2023 Jan 17;18(1):e0271778. doi: 10.1371/journal.pone.0271778 (PMC9844848; doi:10.1371/journal.pone.0271778)
Supplement: S1 Table — (PDF) [file pone.0271778.s002.pdf]

S2 Table. Demographic data, molecular and histology subtypes.

Patient no.	Sex	Age (y)	Molecular subtype	Histology subtype
**1**	F	1	SHH	Desmoplastic/nodular
**2**	F	10	WNT	Classic
**3**	M	8	WNT	Classic
**4**	M	11	nonWNT/nonSHH	Classic
**5**	F	4	nonWNT/nonSHH	Classic
**6**	F	4	nonWNT/nonSHH	Classic
**7**	M	8	nonWNT/nonSHH	Classic
**8**	M	5	nonWNT/nonSHH	Classic
**9**	M	8	nonWNT/nonSHH	Classic
**10**	M	3	nonWNT/nonSHH	Classic
**11**	M	3	nonWNT/nonSHH	Classic
**12**	M	14	nonWNT/nonSHH	Classic
**13**	M	10	nonWNT/nonSHH	Classic
**14**	M	12	nonWNT/nonSHH	Classic
**15**	F	7	nonWNT/nonSHH	Classic
**16**	F	9	nonWNT/nonSHH	Classic
**17**	M	13	nonWNT/nonSHH	Classic
**18**	M	11	nonWNT/nonSHH	Classic
**19**	F	6	nonWNT/nonSHH	Classic
**20**	F	6	nonWNT/nonSHH	Classic

**21**	M	14	nonWNT/nonSHH	Classic
**22**	M	10	nonWNT/nonSHH	Classic
**23**	F	14	nonWNT/nonSHH	Classic
**24**	F	6	Unclassified	Classic

Abbreviation: Y; year, F; Female, M; Male
