# Supplementary figures and images for: Postoperative radiotherapy timing, molecular subgroups and treatment outcomes of Thai pediatric patients with medulloblastoma
Source: PLoS One. 2023 Jan 17;18(1):e0271778. doi: 10.1371/journal.pone.0271778 (PMC9844848; doi:10.1371/journal.pone.0271778)

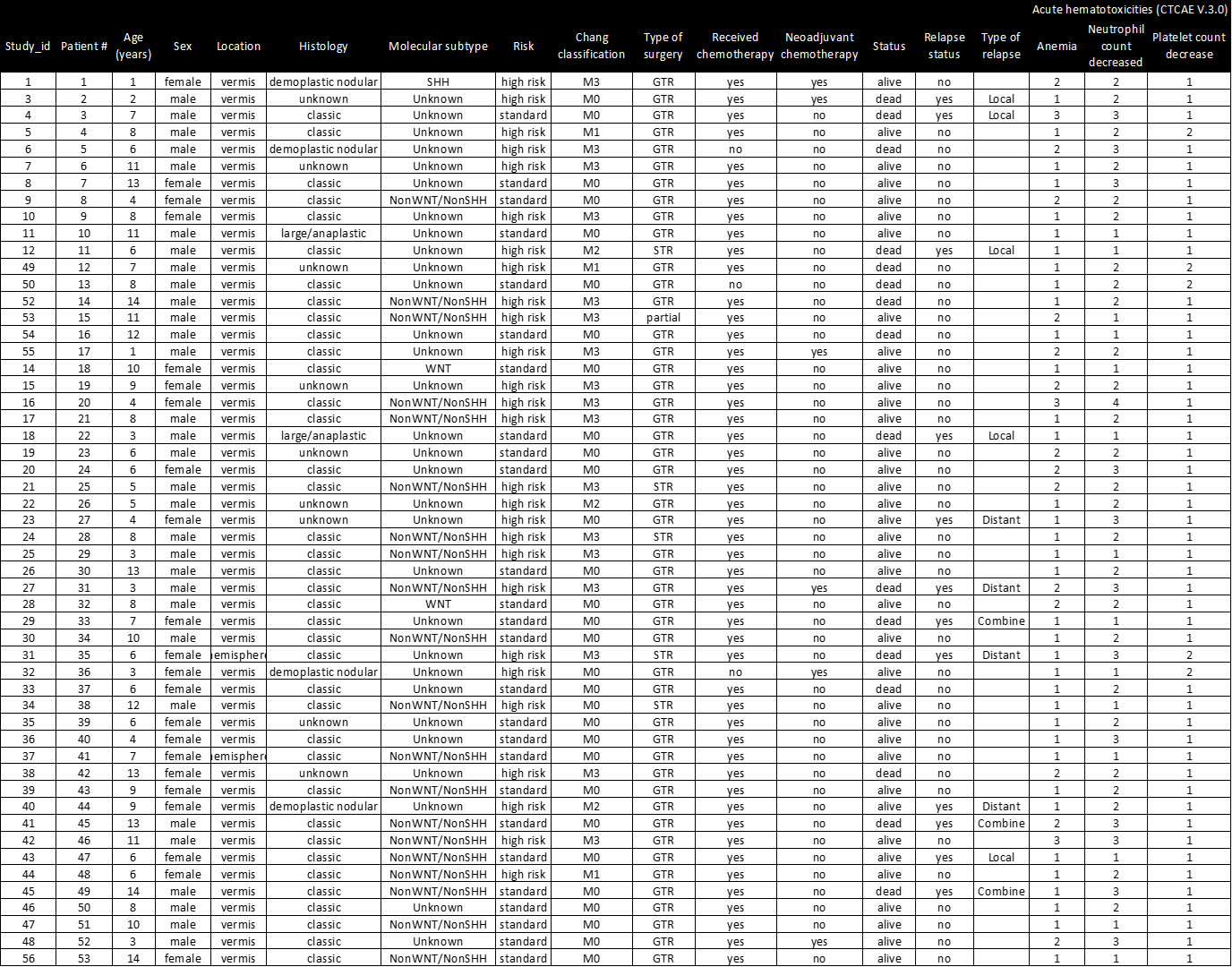

Supplement: S2 Table — (TIF) [file pone.0271778.s003.tif]

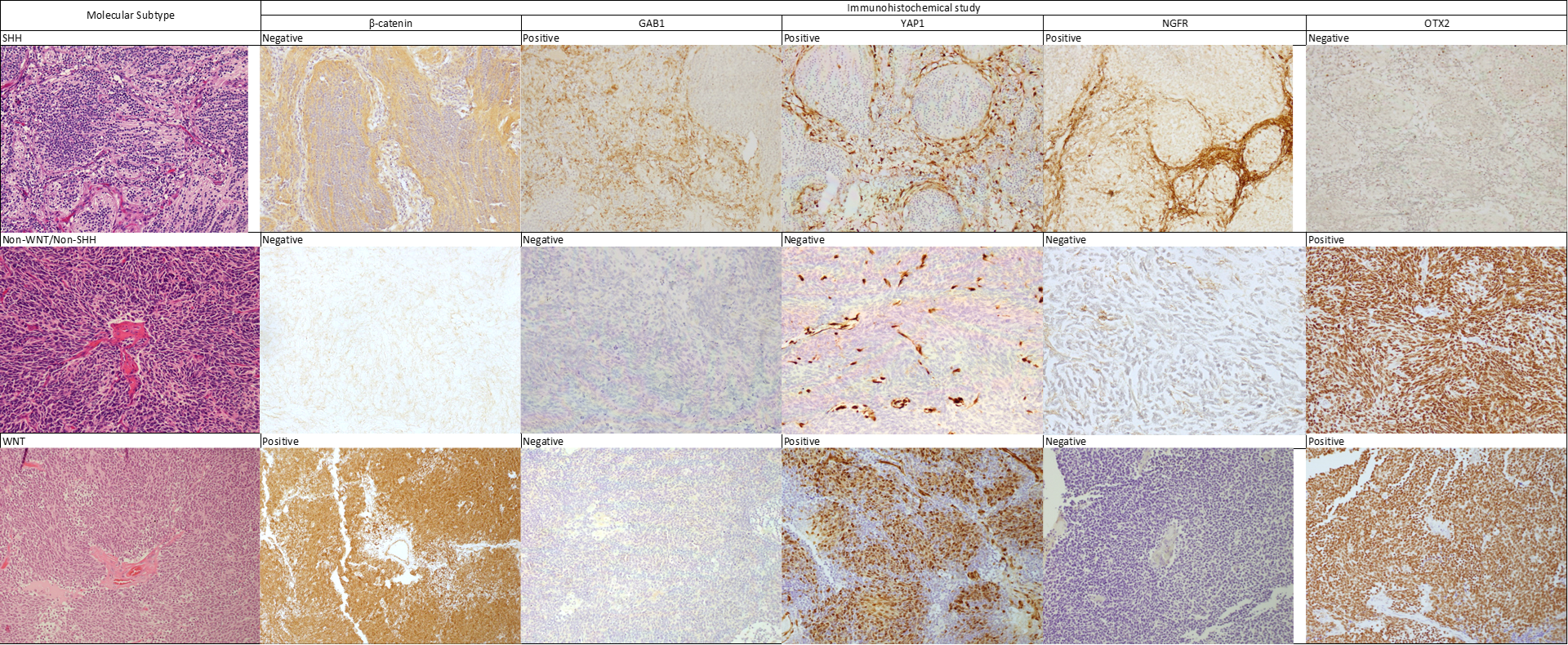

Supplement: S1 Fig — (TIF) [file pone.0271778.s004.tif]
